# Modulating chronic stress

**DOI:** 10.7554/eLife.63996

**Published:** 2020-11-20

**Authors:** Debra A Bangasser, Evelyn Ordoñes Sanchez

**Affiliations:** Department of Psychology and Neuroscience Program, Temple UniversityPhiladelphiaUnited States

**Keywords:** sex differences, stress, social behavior, social dominance, chronic mild stress, principal component analysis, Mouse

## Abstract

Social rank differentially influences how male and female mice respond to chronic stress.

**Related research article** Karamihalev S, Brivio E, Flachskamm C, Stoffel R, Schmidt MV, Chen A. 2020. Social dominance mediates behavioral adaptation to chronic stress in a sex-specific manner. *eLife* **9**:e58723. doi: 10.7554/eLife.58723

Many popular movies focus on high school dramas that feature the classic scenario of an overconfident kid bullying the nerdy victim who, although distraught by their low social status, eventually finds a way to triumph over the bully. As social animals, we find this relatable – our social status affects our lives, and not having the status we want can be stressful and affect our health (Sherman and Mehta, 2020).

Social hierarchies are also common among other mammals. Mice, for example, form complex dominance hierarchies to improve their social stability and reduce conflicts, and can therefore be useful models to study how social rank and biological sex may affect how individuals respond to stress. So far, little is known about how a mouse’s social rank affects its physiology and behavior, and even less about how this differs between sexes. Previous research suggests that the social rank of male mice can influence their health, with subordinate individuals displaying anxiety-like behaviors and greater physiological stress levels (Bartolomucci, 2007). However, female mice also form social hierarchies, and since male and female mice respond differently to stress, it is likely that social rank plays a different role in both sexes (Schuhr, 1987; Williamson et al., 2019; Bangasser and Wicks, 2017).

Now, in eLife, Alon Chen and colleagues at the Max Planck Institute of Psychiatry and the Weizmann Institute of Science – including Stoyo Karamihalev and Elena Brivio as joint first authors – report new insights into a potential link between social status and sex-dependent responses to stress (Karamihalev et al., 2020). Karamihalev et al. developed an automated behavioral monitoring system, the so-called Social Box, to assess social dominance hierarchies of male and female mice living in same sex groups. Then, the researchers evaluated how the social rank affected their responses to stress by measuring physiological and behavioral indicators of stress, activity and anxiety.

First, Karamihalev et al. assessed the hierarchical structure of mice living in groups over four days in the Social Box (Figure 1A) and calculated dominance behavior using a David’s Score, an established method for inferring social hierarchies based on the number and directionality of chases between pairs of mice. Both sexes formed social hierarchies. However, correlations between social rank and the time spent exploring were only found in males, perhaps reflecting sex-specific patrolling behaviors.

**Figure 1.**
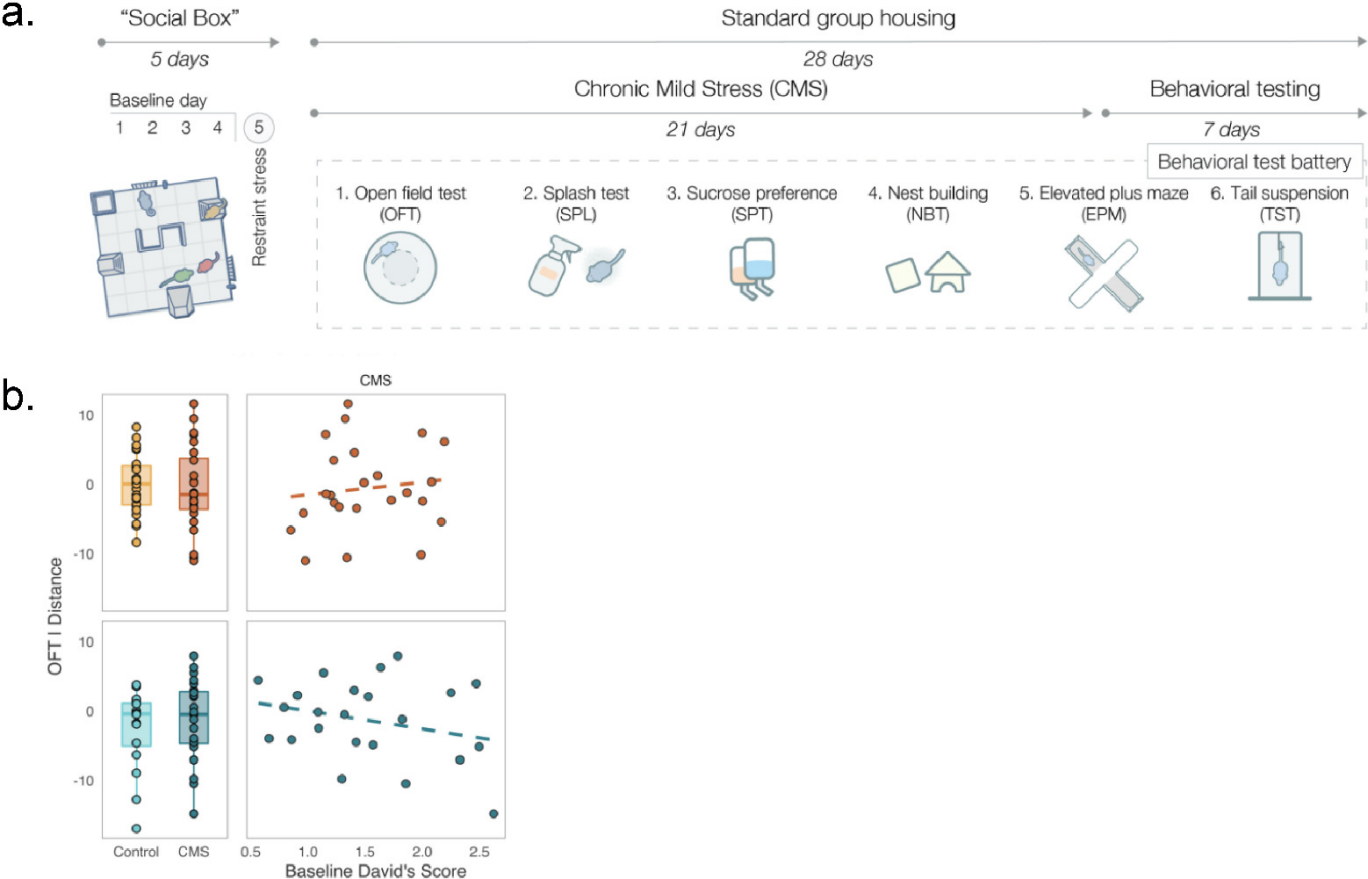
Assessing the effect of social status on chronic mild stress (CMS). (**A**) To study how social rank might influence an individual’s response to chronic stress, Karamihalev et al. kept male and female mice in an automated behavioral monitoring system called the Social Box. The animals lived in same sex groups and were monitored for four days to evaluate their social rank at baseline. On Day 5, mice were subjected to acute stress and then put back in the Social Box to assess social rank. The followinig day, male and female mice were exposed to CMS or standard group housing for 21 days. Following this, all groups of mice had to complete six behavioral tasks, aimed at capturing their activity and aspects of anxiety-like or depression-like behaviors. (**B**) The dominance behavior of male and female mice was evaluated with a David’s Score (a higher score indicates more dominance). An example relationship between social status and one behavior (the distance traveled in the open field test (OFT)) is depicted. The boxplots show the effect of exposure to stress or the control environment for females (top orange and red) or males (bottom turquois and teal). The correlations for CMS mice (right boxes) indicate the opposite relationship between distance traveled and the David’s Score for females and males (boxplots: line indicates median value, scales for behavioral outcomes are relative to female controls).

Next, the researchers examined how mice maintained their social status. The results showed that, for both sexes, the individual in the highest-ranking position did not change often, but the lowest-ranking individual remained consistent only in males. This suggests that females have a less rigid social structure than males. Moreover, exposure to an acute stressor (i.e., restraining the mouse for 15 minutes) did not affect social status in males or females.

Karamihalev et al. then exposed the mice to chronic mild stress for three weeks, which involved subjecting the mice to two random mild stressors per day, such as wet bedding, a tilted cage or overcrowding. The chronically stressed mice’s behavior was compared to that of mice housed in normal conditions. After three weeks, the mice had to complete six behavioral tasks, aimed at capturing their activity and aspects of anxiety-like or depression-like behaviors (Figure 1A). For example, in an open field task, mice were placed into a new environment and their activity (e.g., the distance traveled) as well as anxiety-like behaviors (e.g., the time spent immobile or frozen) were quantified.

After three weeks of being exposed to mild stressors, mice of both sexes lost weight and developed poor-quality coats, indicating that they were indeed stressed. However, mice responded differently to chronic stress depending on their sex and social status. For example, subordinate males subjected to chronic stress displayed more anxious behavior and hyperlocomotion than male mice living without chronic stress, while dominant females exposed to chronic stress were bolder than female mice living stress-free (Figure 1B).

While the social behavior of mice may be less complex than in humans, the findings of Karamihalev et al. nevertheless highlight how social rank can influence an individual’s behavioral response to chronic stress in a sex-specific fashion. These findings can have important implications for future research studying the biological basis of social- and sex-based differences in the stress response. Moreover, the Social Box is an automated method that can determine stable social hierarchies in a relatively short amount of time. If broadly implemented, this method could lead to a better understanding of how social status effects the brain and behavior, and how this differs between sexes. This information could ultimately lead to the development of more targeted treatments for stress-related disorders such as depression or anxiety.
